# Supplementary material for: A Machine Learning Framework for Cancer Prognostics: Integrating Temporal and Immune Gene Dynamics via ARIMA-CNN
Source: Biomedicines. 2025 Nov 11;13(11):2751. doi: 10.3390/biomedicines13112751 (PMC12650626; doi:10.3390/biomedicines13112751)
Supplement: Supplementary file 1 [file biomedicines-13-02751-s001.zip › biomedicines-3965543-supplementary.pdf]

Supplementary Tables

Table S1. Diagnostic Tests

Test	Statistic	Prob.
Ljung-Box (L1) (Q)	0.07	0.78
Jarque-Bera (JB)	1.1	0.58
Heteroskedasticity (H)	1.21	0.42

Table S2. Median Split Analysis of Single Gene.

Immune signatures	HR	95% CI	Cox *p*-value	Log-rank *p*-value
CCL5	0.8638	0.7428-1.0045	0.0572	0.0777
T cells	0.9366	0.8320-1.0544	0.2785	0.1115
B cells	0.8581	0.7673-0.9595	0.0073	0.0087
NK cells	0.9486	0.8175-1.1007	0.4869	0.7471
Granulocyte	1.0565	0.9130-1.2225	0.4605	0.5039
Macrophage	1.0082	0.8026-1.2663	0.9442	0.2526
Th1 cells	0.7485	0.6226-0.8998	0.0020	0.0045
Th2 cells	0.9907	0.8549-1.1481	0.9011	0.5972
CD8 T cells	0.8037	0.7002-0.9224	0.0019	0.0064
PRF1	0.8473	0.7050-1.0182	0.0770	0.2426
GZMB	0.8601	0.7412-0.9980	0.0471	0.2017
Tregs	0.8727	0.7774-0.9796	0.0210	0.0343
MDSCs	1.0723	0.9140-1.2580	0.3915	0.8359

Table S3. CCL5-ARIMA-CNN Extracted Features of Single Gene.

Immune signatures	HR	95% CI	Cox *p*-value	Log-rank *p*-value
T cells	0.9262	0.7931-1.0817	0.3328	0.1115
B cells	0.7322	0.5835-0.9189	0.0072	0.0087
NK cells	0.9278	0.7522-1.1445	0.4842	0.7471
Granulocyte	2.9884	0.1664-53.6804	0.4576	0.5039
Macrophage	1.1190	0.0480-26.1099	0.9442	0.2526
Th1 cells	0.7720	0.6462-0.9222	0.0043	0.0045
Th2 cells	0.9704	0.7959-1.1831	0.7662	0.5972
CD8 T cells	0.7654	0.6450-0.9082	0.0022	0.0064
PRF1	0.8568	0.7165-1.0247	0.0905	0.2426
GZMB	0.8278	0.6810-1.0064	0.0579	0.2017
Tregs	0.5354	0.3088-0.9283	0.0261	0.0343
MDSCs	1.1618	0.7977-1.6920	0.4343	0.8359

Table S4. Hyperparameters and Implementation Details of the Proposed ARIMA-CNN Prognostic Framework/

Hyperparameters & implementation	
**Module**	**Setting**
Data normalization	TPM (per-gene z-score)
Time axis	OS.time (days); one profile per patient
Immune panels	Cytotoxic (CD8, PRF1, GZMB, Th1 markers); Lymphoid (B, Th2, pan-T, NK); Myeloid/Regulatory (granulocyte, Treg, macrophage, MDSC).
ARIMA model selection	Pick by AIC/BIC; confirm with ACF/PACF & Ljung–Box
Final ARIMA for CCL5	ARIMA (5,1,0); residual whiteness satisfied
Forecasting	Single-step recursive out to 500 steps; report mean path + 95% fan
CNN architecture	Conv1D (32, kernel=5, padding='same') → ReLU → BatchNorm → Dropout (0.20) → Conv1D (64, kernel=3, padding='same') → ReLU → GlobalAveragePooling1D → Dense (32, ReLU, L2=1e-4) → Dropout (0.20) → Dense (1, linear)
Training	Loss MSE; Optimizer Adam; batch 32; max 50 epochs; early stopping
Cross-validation	Rolling-origin expanding-window (e.g., 60/70/80/90% train origins), identical splits across all models; seeds fixed
Baselines	ARIMA-only, CNN-only, LASSO–Cox, Random Survival Forest
Primary metrics	Survival: C-index, time-dependent AUC (1/3/5 yr), Integrated Brier Score (IBS); Forecast distribution: 95% coverage & interval width
Secondary (Supplement)	MAE, RMSE, sMAPE; learning curves（train vs. val）; full residual diagnostics
Software	Python (statsmodels ≥0.13; NumPy/Pandas/Matplotlib; TensorFlow/Keras 2.x for CNN); R (survival, survAUC)
Reproducibility	Random seeds fixed; code/config scripts provided; unit labels clarified: Time = OS.time (days); Expression (TPM), z-score

Table S5. Univariate FDR results.

Feature	HR_parsed	CI_low	CI_high	cox_p	cox_q	cox_label
B cells	0.86	0.77	0.96	0.01	0.03	FDR-adjusted
CCL5	0.86	0.74	1.00	0.06	0.13	
CD8 T cells	0.80	0.70	0.92	0.00	0.02	FDR-adjusted
GZMB	0.86	0.74	1.00	0.05	0.13	nominal
Granulocyte	1.06	0.91	1.22	0.46	0.58	
MDSCs	1.07	0.91	1.26	0.39	0.58	
Macrophage	1.01	0.80	1.27	0.94	0.94	
NK cells	0.95	0.82	1.10	0.49	0.58	
PRF1	0.85	0.71	1.02	0.08	0.16	
T cells	0.94	0.83	1.05	0.28	0.50	
Th1 cells	0.75	0.62	0.90	0.00	0.02	FDR-adjusted
Th2 cells	0.99	0.85	1.15	0.90	0.94	
Tregs	0.87	0.78	0.98	0.02	0.08	nominal
B cells	0.73	0.58	0.92	0.01	0.03	FDR-adjusted
CD8 T cells	0.77	0.65	0.91	0.00	0.02	FDR-adjusted
GZMB	0.83	0.68	1.01	0.06	0.13	
Granulocyte	2.99	0.17	53.68	0.46	0.58	
MDSCs	1.16	0.80	1.69	0.43	0.58	
Macrophage	1.12	0.05	26.11	0.94	0.94	
NK cells	0.93	0.75	1.14	0.48	0.58	
PRF1	0.86	0.72	1.02	0.09	0.17	
T cells	0.93	0.79	1.08	0.33	0.55	
Th1 cells	0.77	0.65	0.92	0.00	0.03	FDR-adjusted
Th2 cells	0.97	0.80	1.18	0.77	0.87	
Tregs	0.54	0.31	0.93	0.03	0.08	nominal

Supplementary Figures

(A)

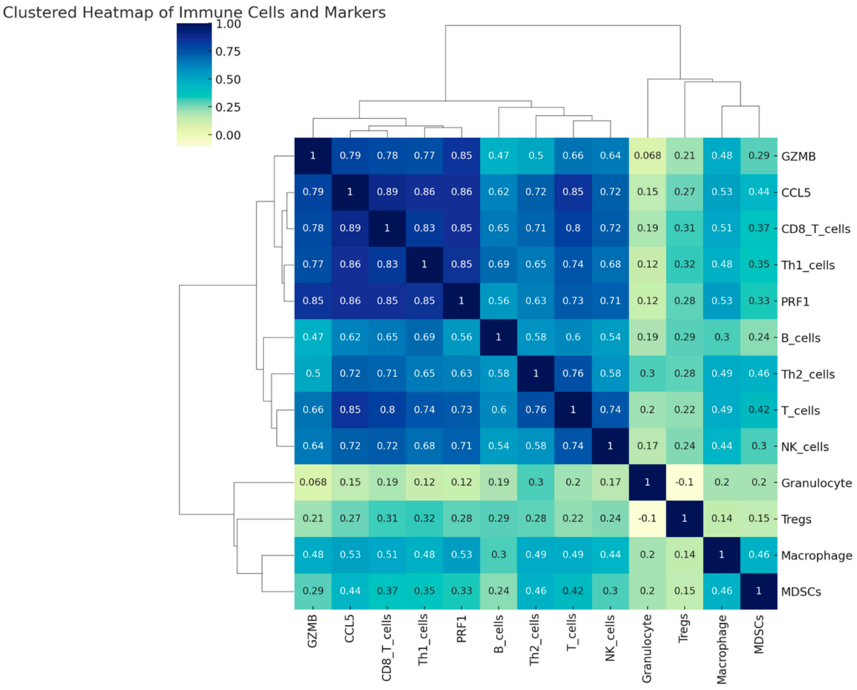

(B)

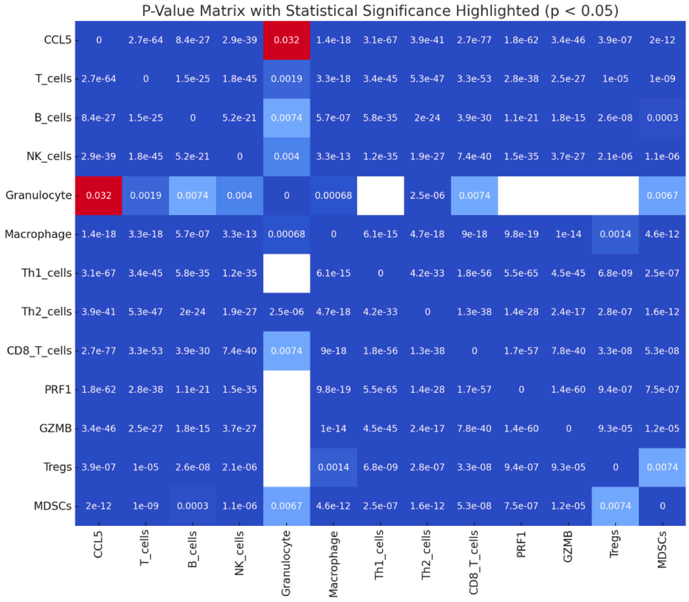

Figure S1. The non-parametric correlation analysis between CCL5 expression and various immune cells.

(A) Non-parametric Spearman rho between CCL5 and the selected immune genes are calculated and analyzed to determine the strength and direction of their linear relationships. (B) The correlation of the *p*-value matrix with incorporated in the figure.

(A)

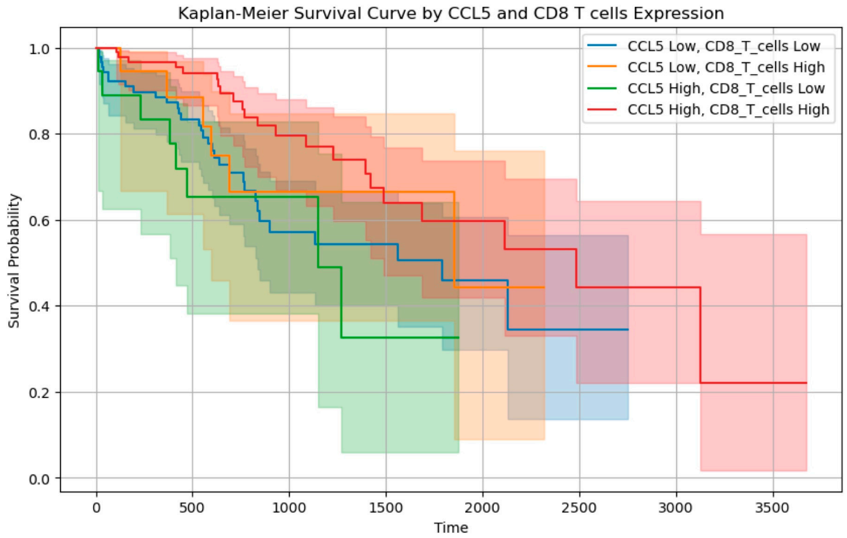

(B)

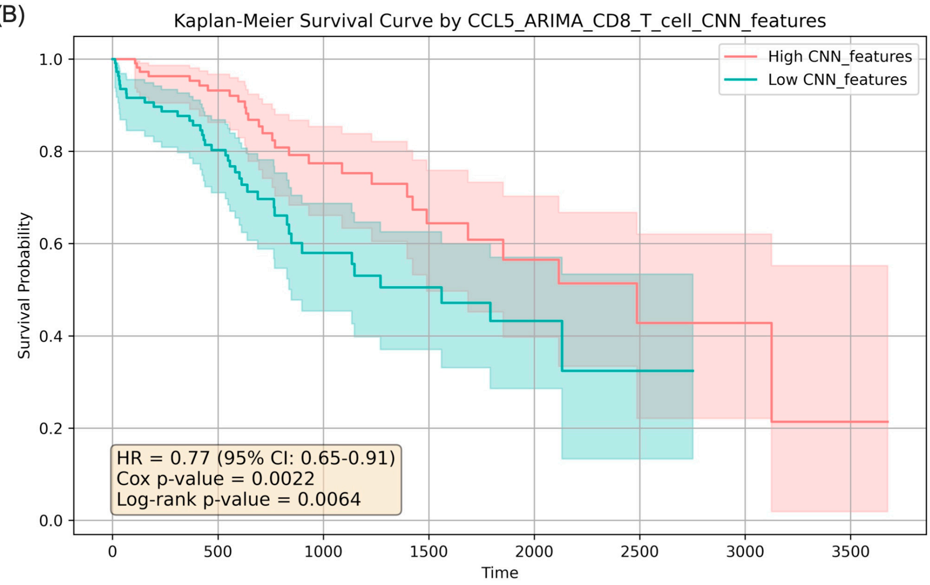

Figure S2. Kaplan-Meier Survival Curve and Cox Regression Analysis for CCL5-ARIMA CD8 T cells CNN-Extracted Features.

(A) The result of the Kaplan-Meier curves graph appears to be more complicated when compared to CCL5 and CD8 T cells, with only two survival curve groups showing significant differences. (B) The ARIMA modeling on CCL5 expression data for CD8 T cells to extract residuals was used as input features for the CNN model alongside gene expression.

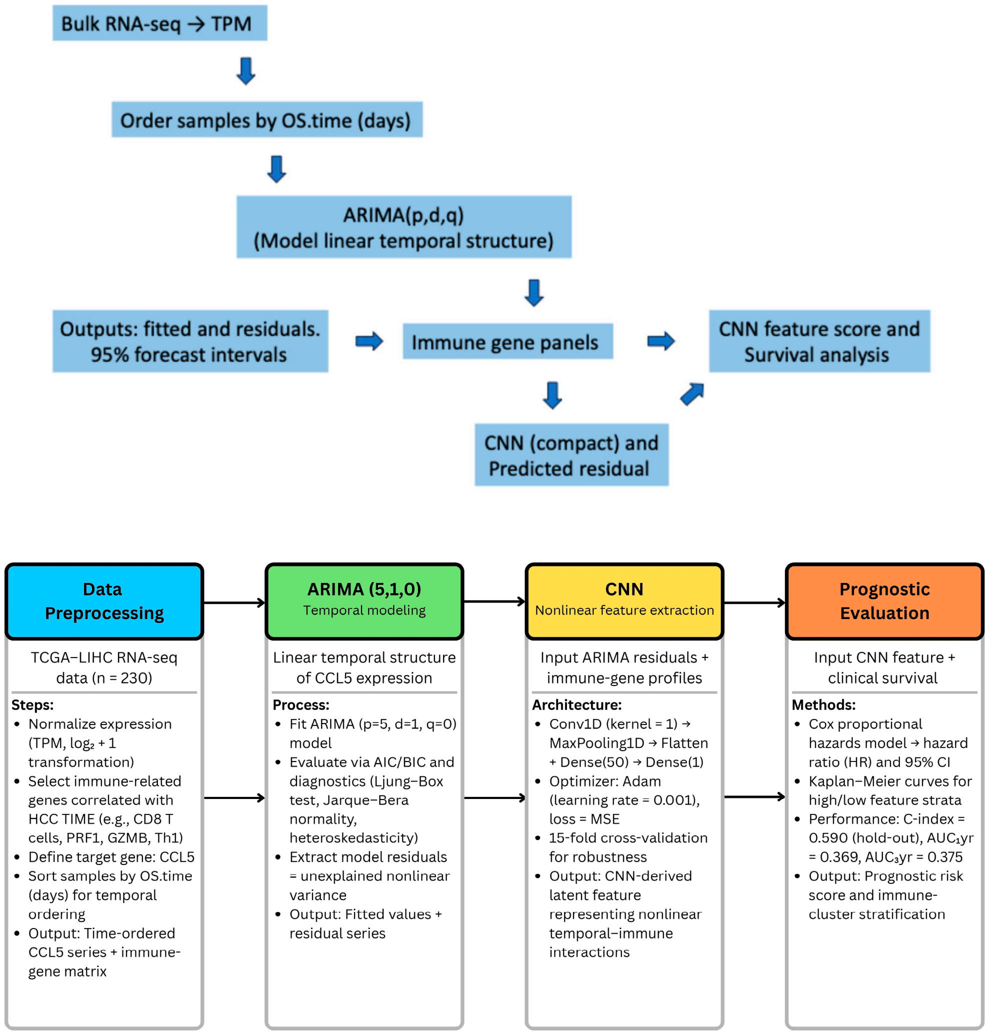

Figure S3. Overview of the ARIMA-CNN prognostic framework.

Bulk RNA-seq is normalized to TPM and samples are ordered by Overall Survival (OS.time; days). ARIMA (p, d, q) models the linear temporal structure of an immune gene and yields residuals/95% forecasts; a compact 1D-CNN learns from these residuals plus curated immune-gene panels to produce a feature score, which is evaluated for prognosis via Cox/Kaplan-Meier. RNA-seq expression (RSEM), OS.time, and OS.event are preprocessed (QC, normalization, sample ordering by OS.time, and train-validation-test split with K-fold cross-validation). A univariate ARIMA (5,1,0) model is fitted to CCL5 to obtain fitted values and residuals. Immune-gene profiles are fed to a 1D-CNN that is trained to approximate the ARIMA residuals; the resulting CNN feature score captures nonlinear temporal-immune structure. The CNN feature score is then entered into a Cox proportional hazards model with OS.time and OS.event to estimate risk. Performance is reported with C-index and time-dependent AUC, with residual diagnostics (ACF/PACF and Ljung-Box) confirming no remaining autocorrelation.
